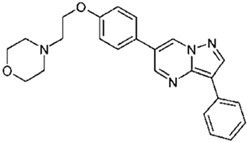# Two Modulators of Skeletal Development: BMPs and Proteoglycans

**DOI:** 10.3390/jdb10020015

**Published:** 2022-04-06

**Authors:** Elham Koosha, B. Frank Eames

**Affiliations:** Department of Anatomy, Physiology and Pharmacology (APP), College of Medicine, University of Saskatchewan, 2D01-107 Wiggins Road, Saskatoon, SK S7N 5E5, Canada; elham.koosha@usask.ca

**Keywords:** proteoglycans, BMPs, cartilage maturation, osteoblast differentiation

## Abstract

During embryogenesis, skeletal development is tightly regulated by locally secreted growth factors that interact with proteoglycans (PGs) in the extracellular matrix (ECM). Bone morphogenetic proteins (BMPs) are multifunctional growth factors that play critical roles in cartilage maturation and bone formation. BMP signals are transduced from plasma membrane receptors to the nucleus through both canonical Smad and noncanonical p38 mitogen-activated protein kinase (MAPK) pathways. BMP signalling is modulated by a variety of endogenous and exogenous molecular mechanisms at different spatiotemporal levels and in both positive and negative manners. As an endogenous example, BMPs undergo extracellular regulation by PGs, which generally regulate the efficiency of ligand-receptor binding. BMP signalling can also be exogenously perturbed by a group of small molecule antagonists, such as dorsomorphin and its derivatives, that selectively bind to and inhibit the intracellular kinase domain of BMP type I receptors. In this review, we present a current understanding of BMPs and PGs functions in cartilage maturation and osteoblast differentiation, highlighting BMP–PG interactions. We also discuss the identification of highly selective small-molecule BMP receptor type I inhibitors. This review aims to shed light on the importance of BMP signalling and PGs in cartilage maturation and bone formation.

## 1. Introduction

Bone morphogenetic proteins (BMPs) are multifunctional growth factors with over 20 family members that play a key role in a variety of biological processes, from embryonic stage to adulthood. However, they are best known for their ability to induce bone formation [1,2,3]. BMPs are the biggest subfamily of the Transforming Growth Factor (TGF-β) superfamily, with highly conserved amino acid sequences from insects to humans [4]. According to sequence and structural homology, BMP ligands have been divided into different groups: BMP-2 and 4; BMP-5, 6, 7, 8, and 8B; BMP-9/GDF-2 (Growth Differentiation Factor-2) and BMP-10; BMP-11/GDF-11 and GDF-8; BMP-12/GDF-7, BMP-13/GDF-6, and BMP-14/GDF-5; BMP-15/GDF-9 and GDF-9b; GDF-1 and 3; and BMP-3 and BMP-3b/GDF-10. BMP family members may form homodimeric or heterodimeric proteins to signal, except for BMP-3 and BMP-15, which biologically act as monomers. However, the osteoinduction ability and signalling activity of homodimers, such as BMP-2, BMP-4, BMP-5, BMP-6, and BMP-7, increase 30- to 50-fold when they are in heteromeric forms, such as BMP2/5, BMP2/6, BMP2/7, and BMP4/7. Higher binding affinity for BMP receptors explain the increased signalling activity of BMP heterodimers [5,6,7].

**Figure 1:**
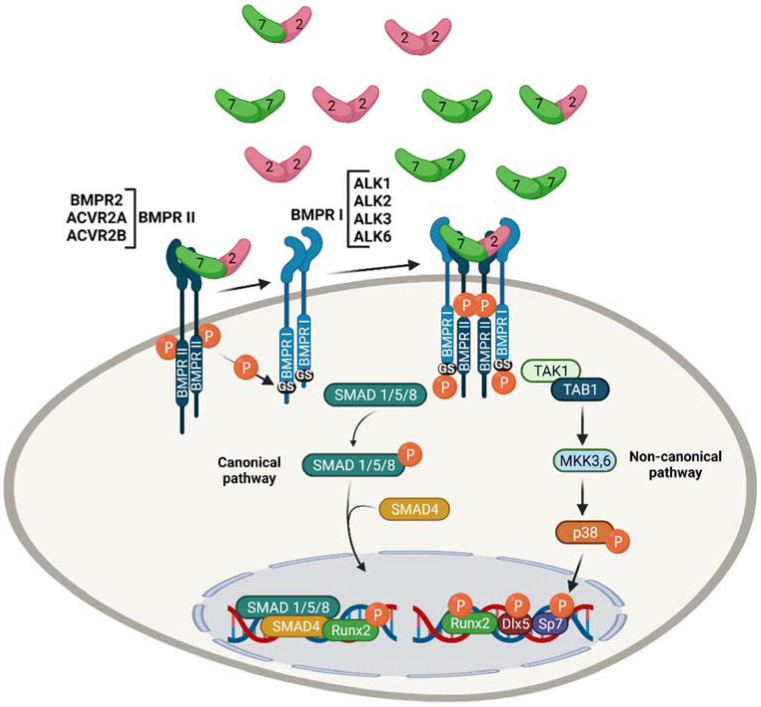
Overview of BMP signalling cascades. BMP homodimers and heterodimers signal through a heterotetrameric complex of serine/threonine kinase BMP type I and type II receptors. In the induced heterotetrameric complex, BMPR type II phosphorylates the GS-domain of BMPR type I to induce canonical and noncanonical BMP signalling pathways. In the canonical pathway, type I receptors phosphorylate Smads 1, 5, or 8 (R-Smad), which form a heteromeric complex with Smad 4, and then translocate to the nucleus. In the nucleus, this complex forms a complex with Runx2 to regulate osteogenic gene expression. The noncanonical signalling cascade, p38 mitogen-activated protein kinase (MAPK), is initiated by TAK1 phosphorylation, which recruits TAB1 and induces the MKK-P38 MAPK signalling pathway. The phosphorylated form of p38 phosphorylates and activates Runx2, Dlx5, and Sp7 transcription factors in the nucleus to initiate the transcription of osteogenic genes. Furthermore, phosphorylated Runx2 promotes the formation of the Smad–Runx2 complex. “Created with BioRender.com (Accessed on 31 January 2022)”.

BMP signalling is mediated through BMP receptors (BMPRs) (Figure 1). A total of four BMP receptors type I (BMPRI) have been identified for BMPs: activin receptor-like kinase 1 (ACVRL1 or ALK1), activin receptor IA (ACVR1 or ALK2), type 1A BMP receptor (BMPRIA or ALK3), and type 1B BMP receptor (BMPRIB or ALK6). Three BMP receptors type II (BMPRII) are known to interact with BMPs: type 2 BMP receptor (BMPRII or BMPR2), activin type 2A receptor (ActRIIA or ACVR2A), and activin type 2B receptor (ActRIIB or ACVR2B). While BMPRIA, BMPRIB, and BMPR2 are specific to BMPs, ALK2, ACVR2A, and ACVR2B are also shared with activins, a member of the TGF-β superfamily [8]. Activin A has a high affinity for the BMPRs type 2, ACVR2A and ACVR2B, which suppresses BMP-6 and BMP-9 signalling by competing for binding to ACVR2A and ACVR2B receptors in conjunction with ALK-2 [9]. BMP dimers bind to BMPRs with varying degrees of affinity [10]. Although TGF-β and activin do not interact with their type I receptors, BMPs can bind to either BMPR I or II on their own; however, their interaction with both receptors increases the ligand’s binding affinity. BMP-9 has the highest affinity for ALK1 and binds poorly to ALK2 [11]; BMP-10 preferentially binds to ALK1, over ALK3 and ALK6; BMP-5, BMP-6, and BMP-7 signal through ALK2, but BMP-6 can also bind to ALK3 and ALK6; and BMP-2 and BMP-4 connect most strongly with ALK3 and ALK6 [12,13]. Interestingly, the different binding affinities of BMPs can determine the mechanism of heterotetrameric signalling complex formation. BMP-6 and BMP-7, for example, bind type II receptors and recruit type I receptors, whereas BMP-2 and BMP-4 primarily bind type I receptors, then recruit type II receptors [14].

BMPs bind to a heterotetrameric complex composed of two BMPRI and two BMPRII transmembrane serine/threonine kinase receptors, acting through them to elicit the downstream transcriptional changes that control cartilage maturation and osteoblast differentiation. BMPs attach to type I and type II receptor complexes, activating the type II receptor, which cross-phosphorylates specific serine and threonine residues at the juxtamembrane glycine-serine-rich (GS) domain of the type I receptor. BMP signalling is transduced intracellularly by a canonical Smad pathway and a noncanonical p38 pathway. In the canonical pathway, activated BMPRs phosphorylate and activate receptor-specific Smad 1, 5, and 8, which bind and recruit Smad 4 (co-Smad) proteins to form heteromeric complexes that translocate into the nucleus. During chondrocyte maturation, BMP-activated Smad complexes control the expression of important osteogenic genes and recruit transcription factors, such as Hoxc-8, FAST-1, OAZ, Runx2, AP-1, and STAT [15,16]. Smad1/Runx2 activity generated by BMP has been demonstrated to upregulate the expression of GADD45β protein, which is present in the nucleus of late hypertrophic chondrocytes and acts as an activator of matrix metalloproteinase-13 (MMP-13) expression. *GADD45β* inhibition slows terminal chondrocyte differentiation [17,18]. In the noncanonical pathway, activated BMPRs phosphorylate TAK1, which recruits TAB1 and initiates a p38 MAPK signalling cascade. In this cascade, the phosphorylated form of p38 travels into the nuclei to regulate gene expression. In pre-osteoblasts, Runx2, Dlx5, and Sp7 are phosphorylated by p38 to increase their transcriptional activity (Figure 1) [19,20,21]. 

Diverse interaction of BMPs with molecules in the extracellular matrix (ECM) is a crucial aspect of their biology. For example, fibrillins, the main structural components of microfibrils, regulate BMP signalling pathways by sequestering them [22,23,24]. Furthermore, *Drosophila* collagen type IV interacts with BMP receptors through binding to Decapentaplegic (Dpp), a functional ortholog of BMP-2 and BMP-4 in vertebrates [25]. Collagen type II features a chordin-like Von Willebrand factor type C (VWC) domain that binds to BMP-2, acting as a negative regulator for this essential chondrogenic growth factor. Many ECM proteins, as well as recognised BMP regulators, have the VWC/chordin domain, which acts as a negative regulator of BMP activities [26]. Moreover, some data suggest that hyaluronan (HA) can mediate and modulate BMP-7 responses [27,28,29]. Another example of the ECM’s participation in BMP biological activities and cell signalling are proteoglycans (PGs). PGs can operate as a coordinator for BMP signalling at the cell surface, where ligands bind to signalling receptors. This review intends to include literature on BMPs, and then PGs, on their individual roles in regulating bone formation, and ends by discussing how these two regulating molecules interact together and how their interaction further modulates skeletal development. 

## 2. Role of BMPs in Skeletal Development

Bone formation, or ossification, begins during the early stages of embryonic life. Intramembranous ossification and endochondral ossification are the two main ways of bone formation. Endochondral ossification, ultimately forming what are called chondral bones, begins when mesenchymal stem cells (MSCs) condense and differentiate into chondrocytes to produce a cartilage template [30,31,32]. A sheath of cells that encapsulates the cartilage differentiates into the perichondrium. Chondrocytes proliferate and produce many matrix molecules, such as collagen type 2 and aggrecan, the latter of which is the most abundant PG in cartilage [33,34,35,36]. Then, a group of chondrocytes, generally in the center region of the developing cartilage, passes through a developmental transition termed maturation. During chondrocyte maturation, cells undergo hypertrophy and express maturation genes, such as Indian hedgehog (*Ihh*) and *col10a1*, while changing and mineralizing their ECM. Cells in the perichondrium surrounding the hypertrophic zone differentiate into osteoblasts at the same time that hypertrophic differentiation begins. Because it ossifies and produces osteoblasts, this area of the perichondrium is called the periosteum [37]. The cartilage ECM they construct is then degraded by invading blood vessels and gradually replaced mostly by marrow, but also by some trabecular bone [38,39].

**Figure 2:**
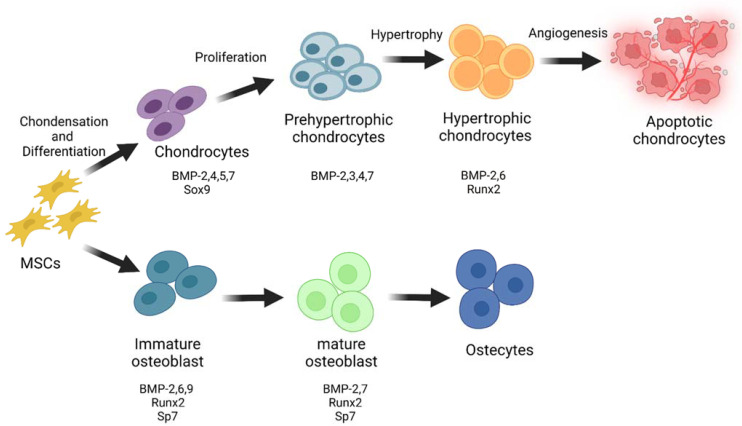
Roles of BMPs in skeletal development. BMP ligands are multifunctional growth factors that are important in a range of biological processes but are best recognised for inducing bone formation. MSCs give rise to chondrocytes, which go through a series of differentiation processes. BMPs govern various phases of chondrocyte differentiation by regulating the expression of Sox9 and Runx2. Sox9 promotes the proliferation and differentiation of MSCs into chondrocytes, while Runx2 induces chondrocyte hypertrophy. Moreover, BMPs promotes osteogenesis by enhancing Runx2 and Sp7 transcription factor activity. “Created with BioRender.com (Accessed on 31 January 2022)”.

One of the key signalling systems involved in cell commitment to the chondrogenic lineage and subsequent progression within the growth plate is the BMP pathway (Figure 2). BMPs control the expression of numerous chondrocyte-specific genes and matrix production and have a role in all stages of chondrogenesis, including early patterning and MSCs condensation, chondrocyte proliferation, and hypertrophic differentiation in the growth plate [40,41]. Multiple BMPs, including BMPs 2, 4, 5, and 7, as well as GDF5, are expressed surrounding or within early condensing mesenchyme in limb buds and developing somites, implying a function for BMP pathways in the early phases of condensation [42,43,44,45,46]. In response to BMP signalling, Sox9, a significant effector of chondrogenesis, induces the production of chondrocytic genes, such as type II collagen [47,48,49]. After the development of a perichondrium, many BMPs, including BMPs 2, 4, and 5, become strongly expressed there [50,51,52,53,54,55]. BMPs 2 and 6 are produced by hypertrophic chondrocytes [52,54]. BMP7 is found in proliferating chondrocytes, especially in the vicinity of the perichondrium [44,54]. Finally, GDFs 5, 6, and 7 are overexpressed in areas where joints are formed. As a result, every area of the growth plate exhibits overlapping expression of several BMP ligands [56,57,58].

BMP signalling induces *Ihh* expression, and both signals cooperate to regulate cartilage maturation [59,60]. Mice missing the *Ihh* gene have significant skeletal defects, including decreased chondrocyte proliferation and maturation, as well as a lack of mature osteoblasts, both of which are deleterious to bone growth [61]. Moreover, Ihh released by maturing chondrocytes induces the perichondrium to ossify [37,59]. During this process, undifferentiated cells in the perichondrium differentiate to the osteoblasts, and cortical bone is formed around the cartilage template. Mouse *Ihh* and zebrafish *ihha* mutants both display delayed perichondral ossification [62,63]. Direct effects on chondrocytes and upregulation of *Ihh* expression are two ways that BMP promotes chondrocytes’ proliferation. According to a study that used a ChIP-based cloning approach, the promoter region of *Ihh* contains numerous motifs that bind to Smad 4 and are essential for BMP-dependent activation [64]. *Ihh*, in turn, keeps BMP levels constant, demonstrating the existence of a positive feedback loop [59,65]. This impact may be direct since Gli transcription factors, which are downstream mediators of Ihh signalling, directly upregulate *BMP-4* and *BMP-7* promoter activity [66].

BMPs are strong osteoblast differentiation and bone formation inducers (Figure 2) [67,68,69]. BMPs maintain bone mass after birth by promoting the differentiation of MSCs into osteoblasts and controlling their differentiation potential [70,71,72,73]. BMP-2, 4, 5, 6, and 7 are known as powerful osteogenic factors [74]. Most BMPs can successfully promote the terminal differentiation of committed osteoblast precursors and osteoblasts; however, BMP-2, 6, and 9 may be the most powerful agents for promoting the osteoblast lineage-specific differentiation of mesenchymal progenitor cells among all of the BMPs investigated [40]. BMP-2 induces or promotes the expression of Runx2 and Sp7 (Osx), which are essential transcription factors for osteoblast differentiation and bone formation, as well as osteoblast differentiation markers, such as alkaline phosphatase (ALP), type I collagen, and osteocalcin [49,75,76,77,78,79,80]. BMP-7 increases matrix mineralization and promotes ALP activity [81,82]. 

## 3. PGs’ Function in Cell-ECM Crosstalk and Skeletal Development

PGs are structural molecules in ECM that offer novel perspectives in cell-ECM crosstalk by regulating the availability of signalling molecules. PGs are sugar-coated proteins that are made up of a core protein, a tetrasaccharide linkage region, and one or more covalently connected repeating disaccharide side chains, which are called glycosaminoglycans (GAGs) [83]. Chondroitin sulfate (CS), heparan sulfate (HS), keratan sulfate (KS), dermatan sulfate (DS), and heparin (HP) are different types of GAGs in PGs’ structure [84]. Together with HA and link proteins, PGs have a tendency to assemble into massive supramolecular complexes > 200 MDa [85]. PGs were thought to be passive, structural molecules, but recent studies have drastically changed that perception by revealing nonstructural, biological roles for PGs through binding and release of numerous signalling molecules and modulating the activity and bioavailability of growth factors and morphogens [86,87].

Although chondrocytes create several minor PGs, aggrecan is the most abundant PG expressed during endochondral bone development [88]. Versican, a large CSPG, is expressed in the early limb bud’s undifferentiated mesenchymal cells and during the beginning of prechondrogenic condensation, before disappearing with chondrocyte differentiation [89]. Concurrently with versican downregulation, a substantial aggrecan expression occurs during the development and maturation of the chondrocyte [90]. The large CSPG aggrecan is the most common PG found in cartilage. Aggrecan includes 100 CS and 25–30 KS chains in adult articular cartilage [91]. The expression pattern of aggrecan mRNA across the growth plate of normal limbs shows that various phases of chondrocyte differentiation need differing amounts of aggrecan in the ECM. The findings that pre-hypertrophic chondrocytes in the wild-type growth plate produce the greatest amounts of aggrecan is consistent with the evidence that the loss of aggrecan across the growth plate impacts chondrocytes during the transition from the pre-hypertrophic to the hypertrophic state [92].

Aggrecan plays a vital role for endochondral bone development and the function of permanent cartilage structures in both human and animal species. Human patients with Kashin–Beck disease, an endemic osteochondropathy found in regions of China, are characterised by low levels of aggrecan, short stature and abnormalities of the limbs and fingers, deformed growth plates, and chondrocyte apoptosis [93,94]. In addition, mutations in the aggrecan coding genes, such as *ACAN* or *AGC1*, are responsible for Spondyloepimetaphyseal dysplasia (SEMD), osteochondritis dissecans with early and severe onset of osteoarthritis in humans, and a variety of short-stature syndromes with rapid bone maturation. *AGC1* mutations induce human aggrecanopathies by causing haploinsufficiency or cartilage structural disturbance [95,96]. Studies on mice have shown that a single 7-bp deletion in exon 5 of the *Agc1* gene results in a premature stop codon in exon 6 and formation of shortened aggrecan molecules which causes cartilage matrix deficit (*cmd*). Homozygous *cmd* mice (*cmd*/*cmd*) exhibited phenotypes including dwarf-like characteristics, chondrodysplasia, aberrant collagen fibrillogenesis, and a cleft palate, which demonstrate aggrecan’s critical role in cartilage maturation. Furthermore, homozygous *cmd* mouse’s articular cartilage was found to have tightly packed chondrocytes surrounded by a little matrix, while the growth plate cartilage had chondrocytes arranged in disorganised columns of diminished length in severely diminished proliferative, pre-hypertrophic zones, consistent with the mouse’s lower proportions [97,98,99,100]. In other studies, in chicks, due to a premature stop codon, chondrocytes produce a truncated aggrecan core protein precursor that is not translocated to the Golgi apparatus for processing, resulting in the absence of aggrecan in cartilage, chondrodysplasia, disrupted organisation of the hyaline and growth plate cartilages, and severe skeletal stature reduction [26,92,101,102,103,104].

**Figure 3:**
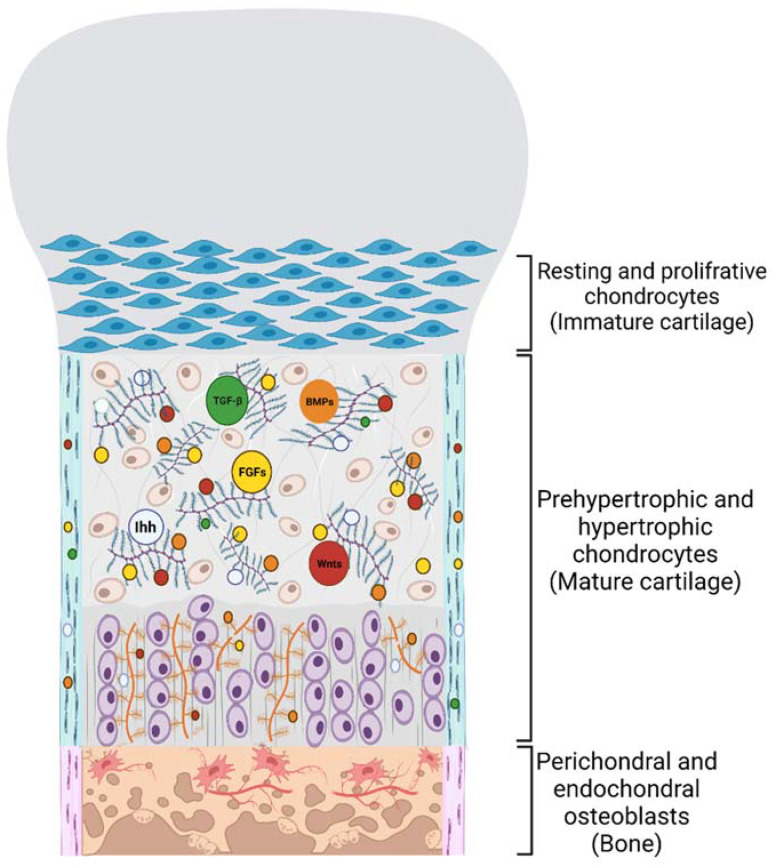
Interactions between PGs and growth factors in the growth plate during endochondral and perichondral ossifications. Chondrocytes are imbedded in a PG-rich ECM so that growth factors can travel in it to regulate cartilage maturation and send a signal to the perichondrium layer to differentiate undifferentiated cells to the osteoblast. Aggrecan is the most abundant CSPG expressed in cartilage, which is required for creating an appropriate Ihh gradient. Furthermore, Wnts, FGFs, BMPs, and TGF-β are among the cell signalling pathways that can be influenced by CSPGs and HSPGs to regulate cartilage maturation and osteoblast differentiation. “Created with BioRender.com (Accessed on 31 January 2022)”.

During endochondral ossification, aggrecan interacts with growth factors and morphogens to control chondrocyte proliferation and differentiation. It was shown that an aggrecan-rich matrix and the correct sulfation of aggrecan’s CS chains are required for the proper interaction with growth factors to establish a morphogen gradient, which modulates the coordination of numerous different signalling pathways during growth plate morphogenesis. Aggrecan is required for creating an appropriate Ihh gradient; these results are supported by the findings that Ihh binds CS chains in vitro and that the Ihh gradient is reduced in the undersulfated CSPG matrix of the brachymorphic mouse growth plate. Furthermore, wingless-related proteins (Wnts) and fibroblast growth factors (FGFs) are among the cell signalling pathways that can be influenced by CS [86,105,106,107]. In another study, a severe chondrodysplasia characterized by the substantial upregulation of TGF-β signalling was revealed in a gene trap mutation in the chondroitin-4-sulfotransferase 1 (*C4st1*) gene, which causes downregulation of 4-O-sulfated chondroitin production [108]. The lack of aggrecan is accompanied by the deregulation of multiple genes previously implicated in hypertrophic chondrocyte development and osteoblast differentiation. The absence of aggrecan in the matrix may also interfere with growth factor availability, which is required for communication between the perichondrium and growing chondrocytes [92]. The early and enhanced invasion of the growth plate hypertrophic zone by blood vessels and osteoblasts may result from an aggrecan-induced matrix deficit; indeed, an anti-angiogenic role for the aggrecan matrix has been suggested [109]. Angiogenic factors, basic fibroblast growth factor (bFGF) and Vascular endothelial growth factor (VEGF), produced by growth plate chondrocytes, induce endothelial cells to move towards hypertrophic cells [39,110]. However, in the lack of aggrecan, a recognised diffusion barrier to several factors in cartilage, this mechanism may be changed (Figure 3) [111].

Perlecan, also known as HSPG-2 (heparan sulfate proteoglycan 2), is a multifunctional, modular PG that promotes chondrocyte proliferation, differentiation, and matrix synthesis by interacting with a wide range of ligands, such as growth factors, morphogens, and ECM-stabilizing glycoproteins. HS, one of the GAGs found in perlecan, is an essential extracellular component. The release of HS-bound cytokines, growth factors, morphogens, proteases, and inhibitory proteins induces matrix remodelling, which regulates numerous cellular pathological and physiological processes. Perlecan possesses chondrogenic capabilities and can influence cell signalling, matrix assembly, and new tissue development via its HS chains. Perlecan’s HS side chains can bind and store growth factors, including FGF-1, 2, 4, and 9, and operate as low-affinity co-receptors, emphasizing the protein’s relevance in growth and development. FGF-7 has also been found to attach to domain III of the perlecan core protein, which appears to control the growth factor’s activity [112]. Perlecan binds, stores, and sequesters FGF-1 in the cartilage matrix, preventing proper Fgfr3 signalling activity and influencing normal cartilage and bone growth. Perlecan’s presence in the pericellular area may help this process. Other HS-containing cell surface receptors may be involved in Fgfr3 signalling, collaborating with perlecan in proper cartilage formation. Moreover, FGF-2 can bind to perlecan and act as a mechanotransducer in chondrocytes. In human articular chondrocytes, FGF-2 can also upregulate the transcription of *matrix metallopeptidases 1* and *13* (*MMP1* and *MMP13*), two enzymes that play significant roles in cartilage degradation. These findings suggest that perlecan plays various roles throughout embryonic development, emphasising the relevance of matrix structure in cellular activities [113,114,115].

In addition to regulating cartilage maturation, PGs have been identified as modulators of osteoblast differentiation [116]. It was shown that CS chains enhance osteoblast differentiation by binding to both cadherin-1 and n-cadherin, decreasing extracellular signal-regulated kinase 1/2 (ERK1/2) phosphorylation, activating Smad 3 and Smad 1/5/8 signalling pathways, and increasing the expression of osteoblastic differentiation markers, such as ALP [117]. Another regulator of osteoblast differentiation are syndecans, which are cell-surface HSPGs that can act as low-affinity co-receptors to help ligands dock and concentrate. Syndecans also influence intracellular signalling by interacting with high-affinity receptors and integrins [118,119]. Out of the four syndecans, Syndecan-2, was found to be particularly associated with osteoblast differentiation during mouse development and in adult bone. Syndecan-2 is also expressed in the periosteum at the start of endochondral ossification, and its expression rises as osteoblast differentiation progresses [120]. BMP-2 and Runx2 are osteogenic mediators that tightly upregulate Syndecan-2 in osteoblast. As a result, the amount of syndecan-2 in osteoblasts appears to be strictly regulated [121,122]. Syndecan-2 overexpression enriches the bone surface with HS and results in an increased bone mass due to a potent inhibition of resorption. Furthermore, it leads to increased bone marrow cell death and lower populations of osteoblast and osteoclast precursors. Multiple pathways, including phosphoinositide 3-kinase (PI3K), MAPK, nuclear factor kappa-B (NF-κB), and protein kinase C, as well as canonical and noncanonical Wnt pathways, are altered by the overexpression of syndecan-2 in osteosarcoma cells. Moreover, it alters the osteoblast environment by downregulating the Wnt/β-catenin/T-cell factor (TCF) pathway [123,124,125]. This family’s syndecan-3 is expressed during limb cartilage development. Syndecan-3 is expressed transiently during the pre-cartilage condensation of the skeletal components of the limb and afterwards in differentiating periosteum osteoblasts. Anti-syndecan-3 antibodies have been demonstrated to decrease limb cartilage development in vitro [126].

Small leucine-rich PGs (SLRPs) are involved in all phases of bone formation, including osteogenesis, mineral deposition, and bone remodeling, by interacting with cell surface receptors and growth factors. Skeletal growth, craniofacial structure, dentin production, and collagen fibrillogenesis are all affected by SLRPs. Biglycan and decorin are both class I subtypes of the SLRPs with CS/DS side chains [127]. Biglycan can regulate the activity of multifunctional growth factors, such as TGF-β, BMP4, and Wnt, which all play a role in the osteogenic program [128,129,130]. Biglycan has been shown to activate the Wnt pathway by binding to Wnt3a, the canonical Wnt ligand, and Wnt receptor low-density lipoprotein receptor-related protein 6 (LRP6). Both glycosylated and non-glycosylated forms of biglycan stimulate this signalling pathway. Biglycan is also implicated in ERK phosphorylation and signal transmission via the transcription factor Runx2. The activation of ERK is mediated by the GAG chains, as phosphorylation of ERK is not identified when only the core protein of biglycan is given [128]. Bone marrow stroma cells (BMSCs) from biglycan/decorin-deficient mice had increased TGF-β signalling due to the inability of biglycan and decorin to sequester TGF-β in the ECM, resulting in a switch in fate from growth to apoptosis. In biglycan/decorin-deficient animals, the early death of osteogenic stem cells and osteoblast precursors resulted in a reduction in the number of mature osteoblasts, contributing to reduced osteogenesis and an osteoporosis-like phenotype. These investigations show that these two SLRPs function in controlling bone mass by modulating the proliferation and survival of osteogenic stem cells via regulating TGF-β activity. Due to a reduction in bone production, mice with a targeted disruption of biglycan develop age-dependent osteoporosis, with smaller trabecular volume and thinner cortices than their wild-type counterparts. Compared to wild-type littermates, biglycan knock-out mice have a considerably reduced capacity to form bone marrow stromal cells (Figure 3) [131].

The relevance of the biological role of PGs in skeletal development has been further demonstrated by using N-Ethyl-N-nitrosourea (ENU) mutagenesis screen that yielded zebrafish mutants that were deficient in cartilage and bone formation. Mutations in two genes, *xylt1* and *fam20b*, were discovered using restriction site associated DNA (RAD) mapping, followed by meiotic mapping and sequencing in a class of mutants with reduced alcian blue staining of PGs in their cartilage matrix [36,132,133]. Xylt1 induces GAG side chain modifications to PG core proteins [134]. Fam20b is a kinase, phosphorylating xylose in the GAG side chain [135]. FAM20B is one of three fam20 family members with sequence similarity 20, also containing FAM20A and FAM20C in mammals. Fam20C is a kinase in this family that phosphorylates hundreds of secreted proteins with a highly conserved Ser-X-Glu/pSer motif. The pseudokinase FAM20A lacks a critical residue for catalysis and forms an evolutionarily conserved homodimer or heterodimer functional complex with FAM20C and activates it. Raine syndrome is caused by FAM20C mutations, which induce bone and craniofacial/dental anomalies, whereas Amelogenesis Imperfecta (AI) is caused by FAM20A mutations. Xylose kinases with a unique active site for binding Gal1-4Xyl1, the initiator disaccharide inside the tetrasaccharide linker region of PGs, are encoded by FAM20B. In HeLa cells, the overexpression of fam20b increases the quantity of HS and CS, whereas fam20b RNA interference diminishes their amount. This essential gene increases the number of GAG chains in PGs by phosphorylating the initiator xylose residue inside the tetrasaccharide linkage region. This phosphorylation is necessary for the elongation of the tetrasaccharide bridge and the assembly of GAG [136]. 

The discovery of zebrafish *xylt1* and *fam20b* mutants with PG synthesis defects, followed by less cartilage matrix and early perichondral bone formation in developing embryos, led to the theory that cartilage PGs impede endochondral ossification. The fact that perichondral bone development begins sooner than wild-type siblings in these mutants indicates a physiologic function for cartilage PGs. Zebrafish with a *fam20b* gene mutation did not create wild-type amounts of CS and HS, typically prevalent in the cartilage matrix [35,36]. Moreover, in *xylt1* and *fam20b* mutant chondrocytes, sox9a expression was reduced, whereas Runx2 transcripts increased. *Ihh*, which is expressed in mature chondrocytes but not in the perichondrium, mediates the inductive event for perichondral bone development. *Ihh* transcripts were upregulated early in *xylt1* and *fam20b* mutant chondrocytes, and genetic epistasis tests revealed that *Ihh* function was required for these PG mutants’ early bone development [36].

## 4. PGs Modulate BMP Signal Transduction

In several in vivo and in vitro settings, BMPs have been discovered to interact with cell-surface and matrix-bound PGs, such perlecan, syndecan, glypican, and betaglycan [137,138]. For example, Xenopus syndecan-1 controls ectoderm dorsoventral patterning by modulating BMP signalling [139]. Glypican-3 has been linked to the modulation of the BMP-4 effects on renal branching morphogenesis [140]. In embryonic kidney explants, glypican-3 loss prevents BMP-2- and BMP-7-dependent ureteric bud formation [141]. In addition, betaglycan, a TGF-β type III receptor, is a membrane HS/CS sulfate PG discovered by its capacity to bind multiple TGF-β family members. Betaglycan can bind a broad spectrum of BMP ligands, including BMP-2, BMP-4, BMP-7, and GDF-5 [142].

**Figure 4:**
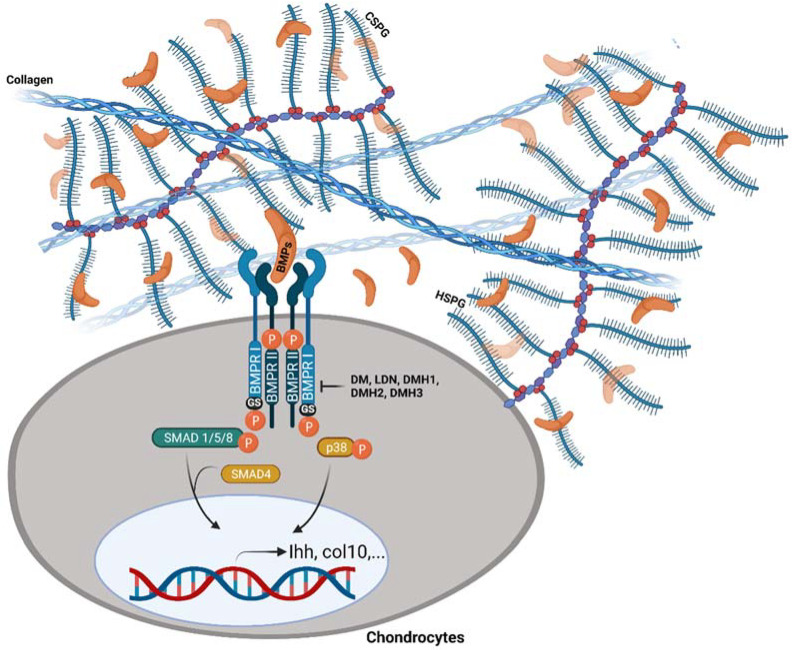
Endogenous and exogenous modulation of BMP signalling transduction by PGs and dorsomorphin analogs. PGs have been shown to regulate the activity or availability of BMPs, either sequestering them from or presenting them to target cell receptors. Dorsomorphin (DM) and its analogs, LDN-193189 (LDN), DMH1, DMH2, and DMH3, specifically antagonize the intracellular kinase domain of BMP type I receptors. “Created with BioRender.com (Accessed on 31 January 2022)”.

PGs may have a key role in skeletal development by regulating BMP signalling (Figure 4). Perlecan works with BMP-2 to increase hypertrophic chondrocyte markers’ expression and osteogenesis [143,144]. Syndecan-3 has been shown to control BMPs activity during chondrogenesis by reducing the effective concentration of BMPs accessible for signalling [126]. Glypican-3 has been linked to the modulation of the BMP-4 effects on vertebrate limb patterning and skeletal development [140]. In vitro, a lack of biglycan inhibits BMP-4-induced osteoblast differentiation due to diminished BMP-4 binding to the receptors, which is totally restored by viral transfection of biglycan [129]. Biglycan has also been discovered to bind directly to BMP-2 and influence BMP-2-induced osteoblast differentiation [145]. In addition, BMP-2 biological activity is modulated by biglycan and decorin, which sequester it in the ECM. In BMSCs from mice with inactivated biglycan and decorin, the expression of BMP-2 signalling components, Smad 1, and phosphorylated Smad 1 were greater than in wild-type cells [131]. In articular chondrocytes, HS deficiency leads to chondrocyte hypertrophy, and enhanced BMP/Smad signalling plays a role in this phenotype [146]. Furthermore, it has been shown that heterozygous loss-of-function mutations in HS synthesis genes, *EXT1* or *EXT2*, which cause multiple hereditary exostoses, a genetic bone condition, results in the upregulation of BMP signalling in the perichondrium. In vitro, BMP signalling is upregulated in Ext1-deficient perichondrium-derived mesenchymal progenitor cells (PDPCs), which leads to increased BMP-induced chondrogenic differentiation [147].

PGs have been discovered to have opposing effects on BMP activity, boosting it in some and decreasing it in others. For instance, HSPGs may either affect BMP activity by functioning as co-receptors, allowing BMPs and their receptors to interact more easily, or serve as a sink for BMPs, preventing them from interacting with BMP receptors, and consequently decrease rather than promote BMP signalling [126,148,149]. *Division abnormally delayed* (*dally*), a *Drosophila* homolog of the glypican family of GPI-linked cell-surface HSPGs, controls cell sensitivity to Dpp signalling by acting as a co-receptor for Dpp [150]. On the other hand, Dally can modulate Dpp distribution by sequestering it, assisting in the morphogen gradient in developing the wing disc [151]. In addition, BMP-4 biological activity is restricted in the Xenopus embryo when it binds to HS chains [152]. In vertebrate, polydactyly and other skeletal anomalies are associated with a lack of cellular response to BMP4 due to a loss-of-function mutation in glypican-3, which leads to Simpson–Golabi–Behmel dysmorphia syndrome in humans and mice [140]. Betaglycan functions as a BMP co-receptor, enhancing BMPs binding to the BMP signalling receptors, ALK3 and ALK6, and hence BMP signalling [142]. In vitro studies on a rat osteoblast cell line, ROS 17/2.8, shows that BMP-7 attaches to HS chains based on their sulfate structures, and the digestion of cell-surface HS hinders BMP-7 binding to the cells, which leads to inhibition of BMP-7-mediated Smad 1/5/8 phosphorylation [153]. In addition, natural BMP antagonists, such as Noggin and Chordin, bind to HS, which enhances their activity. HSPGs that bind to Noggin result in reduced BMP-4 activity [154,155]. Grem1, a member of the Dan family of BMP antagonists that directly binds BMPs to impede signal transduction, can also attach to HSPGs [156].

PG and BMP interactions are predominantly mediated by PG modifications and the domain’s amino acid sequence of BMP ligands. PG modifications affect cell signalling by changing the affinity of ligands and receptors. Sulf1, a secreted sulfatase enzyme, can alter the HSPG structure by removing a sulfate group from the 6-O position of glucosamine in HS chains at the cell surface [157,158,159]. In zebrafish, Sulf1 is an important regulator of BMP signalling that is necessary for appropriate somite development. In knock-down Sulf1 zebrafish embryos, the pharmacological suppression of BMP signalling rescues the development of the horizontal myoseptum and restores the normal migration of the posterior lateral line (PLL) primordium and pigment cells [160]. While HSPGs have well-established functional roles in BMP cell signalling, CSPGs’ participation in this process has often been overlooked. Nevertheless, an analysis of a gene trap mutation in the chondroitin-4-sulfotransferase 1 (*C4st1*) gene that causes the downregulation of 4-O-sulfated chondroitin synthesis leads to a severe chondrodysplasia associated with the downregulation of BMP signalling [108]. On the other hand, domains rich in basic amino acids govern BMP ligands’ distribution and function. One such domain has been found in the n-terminal region of BMP-2 and BMP-4. Changing the basic residues in the n-terminal domains of BMP-2 and BMP-4 inhibits HS binding and changes the mutant proteins’ bioactivity [138,152,161]. Furthermore, a domain with significant HS-binding capacity is in the C-terminal sections of mature BMP-5, BMP-6, and BMP-7. This domain’s amino acid sequence is nearly similar in all three BMPs and has been extremely conserved throughout evolution [162]. 

## 5. Exogenous Mechanisms to Modulate BMP Signalling Pathway

A variety of experimental techniques to modify the BMP signalling pathway have been developed in recent years. According to these findings, BMP signalling can be perturbed by powerful tools and at various molecular levels including: (1) ligand traps and natural soluble antagonists to limit ligand availability to receptors, (2) BMP receptors’ expression or kinase activity suppressors, and (3) intracellular inhibitors [163,164,165,166,167]. In this review, we will concentrate on a group of highly selective small-molecule inhibitors, including dorsomorphin and its analogs, which specifically antagonize the intracellular kinase domain of BMP type I receptors (Figure 4).

Dorsomorphin, also known as Compound C, was the first effective small-molecule BMP type I receptor inhibitor, which was found in a 7500-compound zebrafish library screen based on its ability to disrupt dorsoventral patterning in early zebrafish [168]. Dorsomorphin treatment phenocopies the effect of BMP antagonists, such as Noggin, Chordin, and Follistatin, which induce embryonic dorsalization endogenously. Dorsomorphin’s heterocyclic core structure interacts with the ATP binding site in the kinase domain of BMP type I receptors (Alk2, Alk3, and Alk6) with varying affinities, inhibiting their kinase activity [169,170,171]. The heterocyclic core of dorsomorphin, pyrazolo[1,5-a]pyrimidine core, has been used to synthesize multiple generations of dorsomorphin derivatives, including LDN-193189, DMH1, DMH2, JL5, DMH3, and DMH4. The changes made at the R-positions, R1 and R2, of the pyrazole[1,5-a]pyrimidine core varied amongst analogs of dorsomorphin, resulting in different affinities to the kinase domain of the BMP type I and type II receptors (Table 1) [172].

Based primarily on its effects on zebrafish embryos, LDN-193189 was found as a new compound with enhanced pharmacokinetic characteristics in a structure–activity relationship (SAR) test of dorsomorphin analogs. LDN-193189 has a significantly better selectivity for BMP receptors than dorsomorphin and can be utilised at lower dosages. The signalling activity of the BMP type I receptors ALK2 and ALK3 was effectively suppressed by LDN-193189 [173]. BMP-4 induction of ALP activity is inhibited by LDN-193189 in the C2C12 cell line, which expresses BMP receptors and can be induced to become osteoblasts by BMPs. This indicates that LDN-193189 may influence BMP-induced osteoblast differentiation [174,175]. In another study, LDN-193189 was utilised to demonstrate that overactive BMP receptor signalling contributes to the development of Fibrodysplasia ossificans progressiva (FOP) [176]. FOP is a rare, severe developmental musculoskeletal disease that is caused by activating mutations of the ALK2 gene and marked by heterotopic ossification (HO), endochondral bone development in non-skeletal sites, and a congenital deformity of the great toe [177,178,179]. Intraperitoneal administration of LDN-193189 could partially prevent heterotopic ossification without inducing osteopenia or bone fractures in a mouse model of FOP characterised by a constitutively active version of ALK2 [176]. Furthermore, in Col2a1-Ext1CKO and Fsp1-Ext1CKO, the two multiple hereditary exostoses models, the pharmacological suppression of BMP signalling by LDN-193189 reduces osteochondromagenesis [147]. Both dorsomorphin and LDN-193189 are known to have a variety of “off-target” effects. In addition to inhibiting BMP type I receptor kinase activity, dorsomorphin and LDN-193189 inhibit other cellular kinases, including the TGF-β pathway, AMP-activated kinase (AMPK), receptor tyrosine kinases for platelet-derived growth factor (PDGFR), VEGF, and many other kinases. These off-target effects restrict these drugs’ therapeutic potential and value [180]. The disadvantageous “off-target” effects of dorsomorphin and LDN-193189 were overcome by the identification of dorsomorphin homolog 1 (DMH1), a highly selective small-molecule BMP inhibitor, in a second in vivo zebrafish dorsalization assay with 21 dorsomorphin analogs to generate specific BMP receptor kinase inhibitors. DMH1 inhibits BMP receptors, but not the VEGF pathway, so DMH1 dorsalizes the embryonic axis without disrupting angiogenic processes. Furthermore, DMH1 had no inhibitory effects on TGF-β, activin-induced Smad 2/3 activation, KDR (VEGFR2), ALK5, AMPK, or PDGFR. DMH1 blocks signalling through the ALK2 and ALK3 receptors, with no impact on the ALK6 receptor and no additional side effects [172,181].

DMH2 and DMH3 also belong to this family and are expected to be pan-type I BMP receptor inhibitors with fewer side effects than dorsomorphin and LDN-193189. DMH2 has been reported as the most effective dorsalizing chemical; however, it was less selective than DMH1 and DHM3, since greater doses produced nonspecific developmental effects. JL5 is a DMH2 analog that has better pharmacokinetic features than DMH2. Although all of these BMP inhibitors block BMP type I receptors, JL5 and DMH2 also inhibit BMPR2, while DMH1 and LDN-193189 have no effect on BMPR2 [182,183]. The anti-angiogenic effect of this family’s DMH4 member was elevated, whereas dorsomorphin‘s dorsalization activity was decreased [172]. Dorsomorphin derivatives are inhibitors of BMP signalling pathways and Id family members. Studies have shown that dorsomorphin and DMH1 inhibit BMP-induced Smad 1/5/8 pathway phosphorylation in a dose-dependent manner, while they have no impact on BMP-induced p38 activation [170,172]. However, other studies have shown that all known BMP-induced signallings, canonical and noncanonical pathways, are affected by dorsomorphin and, more effectively, LDN-193189 inhibition of BMP receptors [184]. Moreover, dorsomorphin, DMH1, and DMH2 dramatically reduced Id1, Id2, and Id3 expression, which are the direct mediators of BMP signalling [185]. Other studies have shown reduced BMP-2 and BMP-4 mRNA expression by DMH1, but not ALK2 or ALK3 mRNA expression [186].

## 6. Conclusions and Perspectives

The highly spaciotemporal regulations of skeletal development imply that the mechanisms controlling cell behavior during chondrogenesis and bone formation must be regulated precisely. In this review, we concluded that BMPs and PGs act in concert to orchestrate proper skeletogenesis. Changes in BMP ligands’ availability, as well as PGs’ quantity or structure, can have devastating effects on cartilage maturation and osteoblast differentiation in the embryo. Understanding the BMP signalling system and PGs’ interaction is essential to understanding skeletogenesis mechanisms and for the development of future treatments for osteochondrodysplasias. The data summarized in this review indicate that more research is needed to determine the mechanism of BMPs’ and PGs’ interaction during cartilage maturation, osteoblast differentiation, and its effectiveness for proper skeletal development. Furthermore, while HSPGs have well-established functional roles in BMP cell signalling, CSPGs participation in this process has often been overlooked. In future studies, a greater focus should be given to determine the impact of mutation in PG synthesis genes on the diffusion of BMPs in ECM and their effects in skeletal development. In addition, further research is needed to determine the extent to which BMPs use canonical pathways, noncanonical pathways, or both in different phases of chondrogenesis and bone formation. It will also be intriguing to fathom the situations in which these pathways function synergistically or antagonistically. It is also a paucity of information about the effects of highly selective BMP signalling inhibitors, such as DMH1, on skeletal development. The discovery of BMP signalling inhibitors can pave the way for a deeper understanding of skeletogenesis mechanisms and the development of effective new treatments for skeletal diseases.

## Figures and Tables

**Table 1:** Small-molecule BMP inhibitors: Chemical Names and Structures.

Compound	Chemical Name	Structure
Dorsomorphin	6-[4-[2-(1-Piperidinyl)ethoxy]phenyl]-3-(4-pyridinyl)-pyrazolo[1,5-a]pyrimidine	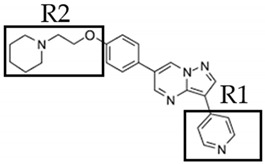
LDN-193189	4-[6-[4-(1-Piperazinyl)phenyl]pyrazolo[1,5-a]pyrimidin-3-yl]-quinoline dihydrochloride	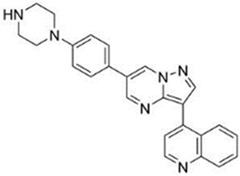
DMH1	4-[6-(4-Propan-2-yloxyphenyl)pyrazolo[1,5-a]pyrimidin-3-yl]quinoline	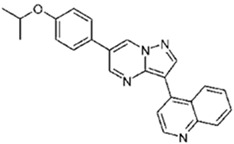
DMH2	4-(2-(4-(3-(quinolin-4-yl)pyrazolo[1,5-a]pyrimidin-6-yl)phenoxy)ethyl)morpholine	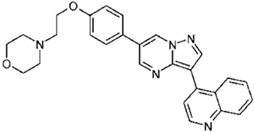
JL5	4-(3-(4-(3-(quinolin-4-yl)pyrazolo[1,5-a]pyrimidin-6-yl)phenyl)propyl)morpholine	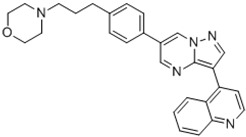
DMH3	N,N-dimethyl-3-(4-(3-(quinolin-4-yl)pyrazolo[1,5-a]pyrimidin-6-yl)phenoxy)propan-1-amine	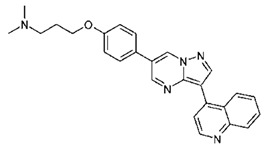
DMH4	4-(2-(4-(3-phenylpyrazolo[1,5-a]pyrimidin-6-yl)phenoxy)ethyl)morpholine